# K-variant *BCHE* and pesticide exposure: Gene-environment interactions in a case–control study of Parkinson’s disease in Egypt

**DOI:** 10.1038/s41598-018-35003-4

**Published:** 2018-11-08

**Authors:** Thomas W. Rösler, Mohamed Salama, Ali S. Shalash, Eman M. Khedr, Abdelhalim El-Tantawy, Gharib Fawi, Amal El-Motayam, Ehab El-Seidy, Mohamed El-Sherif, Mohamed El-Gamal, Mohamed Moharram, Mohammad El-Kattan, Muhammad Abdel-Naby, Samia Ashour, Ulrich Müller, Astrid Dempfle, Gregor Kuhlenbäumer, Günter U. Höglinger

**Affiliations:** 1 German Center for Neurodegenerative Diseases (DZNE), Munich, Germany; 2 Department of Neurology, Technical University of Munich, Munich, Germany; 3 Medical Experimental Research Center (MERC), Mansoura University, Mansoura, Egypt; 4 Toxicology Department, Mansoura University, Mansoura, Egypt; 5 Department of Neurology, Ain Shams University, Cairo, Egypt; 6 Department of Neurology, Assiut University, Assiut, Egypt; 7 Department of Neurology, Mansoura University, Mansoura, Egypt; 8 Department of Neurology, Sohag University, Sohag, Egypt; 9 Department of Neurology, Zagazig University, Zagazig, Egypt; 10 Department of Neurology, Tanta University, Tanta, Egypt; 11 Institute for Human Genetics, Justus Liebig University Giessen, Giessen, Germany; 12 Institute of Medical Informatics and Statistics, Kiel University, Kiel, Germany; 13 Department of Neurology, Kiel University, Kiel, Germany; 14 Munich Cluster for Systems Neurology (SyNergy), Ludwig-Maximilians-University, Munich, Germany

## Abstract

Pesticide exposure is associated with increased risk of Parkinson’s disease (PD). We investigated in Egypt whether common variants in genes involved in pesticide detoxification or transport might modify the risk of PD evoked by pesticide exposure. We recruited 416 PD patients and 445 controls. Information on environmental factors was collected by questionnaire-based structured interviews. Candidate single-nucleotide polymorphisms (SNPs) in 15 pesticide-related genes were genotyped. We analyzed the influence of environmental factors and SNPs as well as the interaction of pesticide exposure and SNPs on the risk of PD. The risk of PD was reduced by coffee consumption [OR = 0.63, 95% CI: 0.43–0.90, *P* = 0.013] and increased by pesticide exposure [OR = 7.09, 95% CI: 1.12–44.01, *P* = 0.036]. The SNP rs1126680 in the butyrylcholinesterase gene *BCHE* reduced the risk of PD irrespective of pesticide exposure [OR = 0.38, 95% CI: 0.20–0.70, *P* = 0.002]. The SNP rs1803274, defining K-variant *BCHE*, interacted significantly with pesticide exposure (*P* = 0.007) and increased the risk of PD only in pesticide-exposed individuals [OR = 2.49, 95% CI: 1.50–4.19, *P* = 0.0005]. The K-variant *BCHE* reduces serum activity of butyrylcholinesterase, a known bioscavenger for pesticides. Individuals with K-variant *BCHE* appear to have an increased risk for PD when exposed to pesticides.

## Introduction

Parkinson’s disease (PD) is a progressive neurodegenerative disease with intraneuronal aggregation of alpha-synuclein, and characteristic motor and non-motor symptoms, affecting more than 6.2 million people globally^1^. Monogenic inheritance only accounts for a small proportion of PD cases, whereas the etiology in more than 90% of the patients appears as complex interplay of multiple genetic and environmental risk factors^2^. Knowledge about causative factors is of utmost relevance to develop preventive measures and disease-modifying therapies.

In the 1980s, 1-methyl-4-phenylpyridinium (MPP^+^) was discovered to induce neurodegeneration and parkinsonism in drug abusers^3^. MPP^+^ was marketed as pesticide under the tradename cyperquat^4^ and showed structural similarities to other known pesticides, e.g. paraquat. This finding triggered intensive research about potential links between pesticides and PD. Three meta-analyses of epidemiological studies investigating the association of pesticides and PD have been carried out so far^5,6^. They consistently concluded that pesticide exposure and factors related to pesticide exposure (e.g. rural living, farming or well water drinking) are positively associated with the risk to develop PD^5,6^.

Recent studies investigated whether risk conferred by pesticide exposure is modified by single nucleotide polymorphisms (SNPs) in candidate genes involved in detoxification or neuronal uptake of pesticides (e.g. aldehyde dehydrogenase 2 (*ALDH2*)^7^, cytochrome P450 2 D6 (*CYP2D6*)^8^, manganese-dependent superoxide dismutase (*MnSOD*)^9^, nitric oxide synthase 1 (*NOS1*)^10^, NAD(P)H dehydrogenase [quinone] 1 (*NQO1*)^9^, multidrug resistance protein 1 (*MDR1*)^11^, glutathione-S-transferase (*GST*)^12^, paraoxonase 1 (*PON1*)^13^, dopamine transporter (*SLC6A3*)^14^. Some studies found an interaction of pesticide exposure with genetic variants in *ALDH2*^7^, *CYP2D6*^8^, *NOS1*^10^, *PON1* concerning PD risk^13^. Most studies, however, did not find significant gene-environment interactions. A recent study searched genome-wide for genetic modifiers of PD risk conferred by pesticides in a relatively small number of patients, without finding any significant results^15^.

In Egypt, pesticides are used extensively and under low safety standards, including types of pesticides that have been banned in Western countries for many years due to safety concerns^16^. Presently, around 40% of the Egyptian workforce is employed in agriculture with high likeliness of pesticide exposure^17^. Furthermore, Egypt has an age-adjusted prevalence (≥50 years) of 2,500-2,750 PD cases per 100,000 in distinct governorates^18,19^, with a three-fold excess in rural over urban residence, which represents a massive increase by international comparison, particularly also compared to surrounding Arab countries^20^. Therefore, we collected a case-control sample in Egypt to study the association of PD with exposure to pesticides and their interaction with genetic variants involved in pesticide metabolism. Genes and variants of interest were selected by a detailed literature research on genes important for pesticide detoxification with a possible relation to neurodegeneration.

## Results

### Participants’ characteristics

The study sample consisted of *n* = 416 unrelated PD patients and *n* = 445 unrelated healthy controls of Egyptian ancestry (Table 1). The sex distribution did not differ between the groups, but PD patients were older than controls. Age at diagnosis, disease duration, and Hoehn and Yahr stage distribution for the PD patients are shown in Table 1.

**Table 1:** Participants’ characteristics.

Characteristic	PD cases (*n* = 416)	Controls (*n* = 445)	OR	95% CI	*P*-Value
Sex (male) [*n* (%)]	253 (60.8)	263 (59.1)	1.074	(0.82–1.41)	0.608
Age (y) [*mean* (SD)]	58.4 (8.8)	48.6 (11.8)	2.56*	(2.18–3.02)	<0.001*
Age at diagnosis (y) [*mean* (SD)]	55.0 (8.8)	n.a.			
Disease duration (y) [*mean* (SD)]	3.5 (2.8)	n.a.			
Hoehn & Yahr stage					
Stage 1 [*n* (%)]	121 (29.1)	n.a.			
Stage 1.5 [*n* (%)]	111 (26.7)	n.a.			
Stage 2 [*n* (%)]	107 (25.7)	n.a.			
Stage 2.5 [*n* (%)]	20 (4.8)	n.a.			
Stage 3 [*n* (%)]	40 (9.6)	n.a.			
Stage 4 [*n* (%)]	14 (3.4)	n.a.			
Stage 5 [*n* (%)]	3 (0.7)	n.a.			
Head trauma [*n* (%)]	10(2.4)	19 (4.3)	0.55	(0.24–1.17)	0.135
Rural living					
Most of life [*n* (%)]	142 (34.1)	135 (30.3)	1.19	(0.89–1.59)	0.233
Childhood [*n* (%)]	138 (33.2)	135 (30.3)	1.14	(0.86–1.52)	0.372
Well water drinking [*n* (%)]	27 (6.5)	0 (0.0)	n.a.	n.a.	<0.001^‡^
Illiteracy [*n* (%)]	129 (31.0)	100 (22.5)	1.55	(1.14–2.11)	0.005
Coffee consumption [*n* (%)]	66 (15.9)	134 (30.1)	0.44	(0.31–0.61)	<0.001
Black tea consumption [*n* (%)]	391 (94.0)	418 (93.9)	1.01	(0.58–1.77)	0.972
Cigarette smoking [*n* (%)]	69 (16.6)	78 (17.5)	0.94	(0.65–1.33)	0.714
Shisha smoking [*n* (%)]	39 (9.4)	31 (7.0)	1.38	(0.85–2.27)	0.198
Pesticide exposure^#^	163 (39.2)	148 (33.2)	1.29	(0.98 1.71)	0.071
Use of pesticides vs. non-exposed					
At home only [*n* (%)]	44 (14.8)	48 (13.9)	1.08	(0.69–1.68)	0.745
At work [*n* (%)]	87 (25.6)	69 (18.8)	1.48	(1.04–2.12)	0.032
Pesticide classes used at home or/and at work vs. non-exposed					
Insecticides only [*n* (%)]	82 (24.5)	104 (25.9)	0.93	(0.66–1.29)	0.926
Herbicides and other [*n* (%)]	46 (15.4)	11 (3.6)	4.91	(2.58–9.94)	<0.001

Odds ratio (OR), 95% confidence interval (CI) and *P*-value were calculated using logistic regression; *OR and CI calculated by logistic regression analysis for the ~10-year age difference between cases and controls. ^#^Ever use of pesticides at home or at work, or >50% lifetime residence in rural areas. ^‡^Calculated using Fisher’s exact test because the logistic regression did not converge. The odds ratio for well water drinking could not be calculated because none of the controls drank well water. n.a., not applicable.

### Environmental factors affect the risk of PD

In a first exploratory comparison, we analyzed differences in single environmental factors between PD patients and control individuals (Table 1). Coffee consumption was the only factor associated with a decreased risk of PD. Factors associated with increased risk of PD were age, well water drinking, illiteracy, use of pesticides at work, and specifically the use of herbicides at home and/or at work. The use of insecticides at home and/or at work showed a trend towards a positive association with PD.

We then constructed a logistic regression model to assess the composite influence of environmental factors on the risk for PD. We considered all factors, which were significantly associated with PD in the single factor analysis (Table 1) for the model.

Well water drinking was significantly associated with PD, but 25 out of 27 well water drinkers (93%) were also exposed to pesticides, leading to high collinearity (*P* < 0.001) between these factors, with pesticide exposure rather than well water drinking being the likely causal risk factor for PD^2,5^. Illiteracy was also highly collinear with pesticide exposure (*P* < 0.001), with 85% of the illiterate participants being pesticide exposed. Again, pesticide exposure rather than illiteracy was the biologically plausible risk factor^2,5^. Therefore, well water drinking and illiteracy were excluded from the logistic regression model.

In our cohort, 79% of the coffee drinkers were not pesticide-exposed. Thus, coffee consumption was inversely correlated with pesticide exposure (*P* < 0.001). Nevertheless, we included coffee in the regression model because it is a well-known protective factor for PD^2,5^. Age was expectedly a highly significant risk factor for PD (*P* < 0.001). In addition, age interacted with pesticide exposure (*P* = 0.031) as explanatory variables for PD risk. Age was a somewhat stronger risk factor in the pesticide exposed subgroup (OR for 10 years age difference in the exposed subgroup: 2.95, 95% confidence interval: 2.39–3.71; in the unexposed subgroup: 2.56, 95% confidence interval: 1.18–3.02).

Consequently, the final logistic regression model contained pesticide exposure, coffee consumption, age, and the interaction (age x pesticide exposure). In this analysis, age and pesticide exposure were confirmed as significant risk factors for PD, while coffee consumption was protective (Table 2).

**Table 2:** Environmental factors affecting the risk for PD.

Variable	OR	95% CI	*P-*Value
Age	2.86^+^	(2.31–3.61)	2 × 10^−16^
Coffee consumption	0.63	(0.43–0.90)	0.013
Pesticide exposure*	7.09	(1.12–44.01)	0.036

Statistical analysis was carried out by logistic regression (see results section for details of the model) using the model formula: affection status ~ pesticide exposure + coffee + age + (age ∗ pesticide exposure); ^+^OR and CI calculated by logistic regression analysis for the ~10-year age difference between cases and controls. *Ever use of pesticides at home or at work, or >50% lifetime residence in rural areas.

### Influence of protective measures on PD risk

To identify factors modulating the risk for PD caused by occupational pesticide exposure, we compared protective measures in the subgroups of occupationally exposed participants, all of whom worked with pesticides in agriculture (*n* = 156 overall, *n* = 87 PD, *n* = 69 controls). Most of them had worked for more than 20 years with pesticides (90.8% of PD patients, 91.3% of controls). Their risk for PD was significantly reduced by wearing gloves during work and by washing hands after work, but not by changing clothes and taking a shower after work (Table 3).

**Table 3:** Influence of protective measures on the risk for PD.

Protective measure	PD cases (*n* = 87)	Controls (*n* = 69)	OR	95% CI	*P-*Value
Wearing gloves during work					
Yes [*n* (%)]	33 (37.9)	44 (63.8)	0.35	(0.18–0.66)	0.002
No [*n* (%)]	54 (62.1)	25 (36.2)			
Washing hands after work					
Yes [*n* (%)]	67 (77.0)	66 (95.7)	0.15	(0.03–0.47)	0.003
No [*n* (%)]	20 (23.0)	3 (4.3)			
Changing clothes after work					
Yes [*n* (%)]	17 (19.5)	13 (18.8)	1.05	(0.47–2.37)	0.912
No [*n* (%)]	70 (80.5)	56 (81.2)			
Shower after work					
Yes [*n* (%)]	10 (11.5)	15 (21.7)	0.47	(0.19–1.07)	0.088
No [*n* (%)]	77 (88.5)	54 (78.3)			

Odds ratio (OR), 95% confidence interval (CI) and *P-* value were calculated using logistic regression.

### Effect of variants in *BCHE* on PD risk

Next, we investigated the influence of genetic factors and gene-environment interactions on the risk to develop PD. After marker- and sample-wise quality control of the genotyping data, *n* = 372 PD patients and *n* = 394 control individuals remained, of whom *n* = 275 (*n* = 147 PD, *n* = 128 controls) had been exposed to pesticides. We expanded the logistic regression model described above by the SNP data and by an interaction term (SNP × pesticide exposure). We analyzed the dominant model for all SNPs (Supplementary Table S1).

Only the SNP rs1126680 in the butyrylcholinesterase gene (*BCHE)* showed significant association with PD *per se* (Table 4; minor allele (G) frequency 0.140 in controls, 0.060 in PD; *P* = 0.007, OR = 0.38, 95% confidence interval: 0.20–0.70).

**Table 4:** Variants in *BCHE* affecting the risk for PD and their interaction with pesticide exposure.

SNP	Model	SNP main effect			Interaction
		OR	95% CI	*P-*Value	*P-*Value
Complete sample – pesticide exposed vs. non-exposed					
rs1126680	dominant	0.38	(0.20–0.70)	0.002	0.795
rs1803274	dominant	0.75	(0.49–1.15)	0.187	0.007
Insecticide exposed subgroup vs. non-exposed					
rs1803274	dominant	n.a.	n.a.	n.a.	0.002
Herbicide exposed subgroup vs. non-exposed					
rs1803274	dominant	n.a.	n.a.	n.a.	0.893

Logistic regression analysis of SNP main effect and interaction with pesticides. The interaction *P*-value is derived from the interaction term between SNP and pesticide exposure. The logistic model contained SNP, pesticide exposure, age, coffee consumption and the interaction between pesticides and SNP as well as pesticides and age; affection status ~ SNP + pesticide exposure + coffee + age + (SNP ∗ pesticide exposure) + (age ∗ pesticide exposure); n.a. not applicable for subgroup analysis.

**Figure 1:**
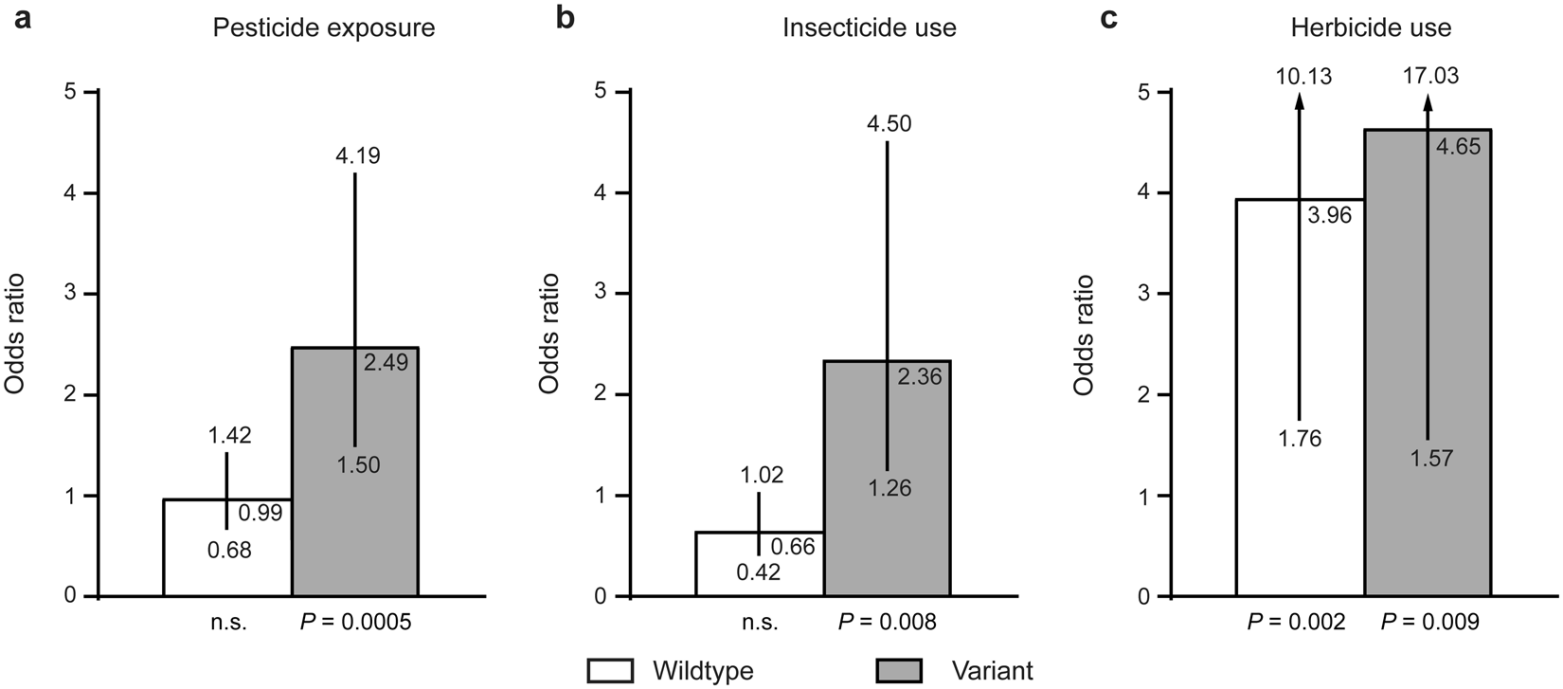
The SNP rs1803274 in *BCHE* increases the risk for PD in pesticide-exposed individuals. Effect of pesticide exposure on PD risk per genotype in the dominant model. The statistical measures are reported with reference to the same genotype (wt = wildtype/wildtype), (var = wildtype/variant or variant/variant) without pesticide exposure for which the odds-ratio is per definition 1. Odds-ratios are found in the upper right corner of the bars and 95% confidence intervals of the odds-ratios are indicated below and above the error bars. (**a**) Analysis of the whole sample comparing pesticide exposed to unexposed individuals. (**b**) Analysis of the subgroup of insecticide only exposed subgroup comparing insecticide exposed to unexposed individuals. (**c**) Analysis of the subgroup of herbicide and other pesticide exposed individuals comparing this group to unexposed individuals.

SNP rs1803274 was not associated with PD *per se* (minor allele (A) frequency 0.241 in controls, 0.250 in PD), but interacted significantly with pesticide exposure (Table 4; interaction: *P* = 0.007 dominant). In carriers of the minor allele of rs1803274, pesticide exposure significantly increased the risk of PD (Fig. 1a; *P* = 0.0005, OR = 2.49, 95% confidence interval: 1.50–4.19) compared to unexposed individuals with the same genotype.

In addition, we performed a subgroup analysis comparing non-exposed participants specifically to participants exposed only to insecticides or to herbicides, respectively. The interaction of rs1803274 was significant in the insecticide only exposed subgroup (Table 4; *P* interaction = 0.002), but not in the herbicide-exposed subgroup (Table 4; *P* interaction = 0.893). In carriers of the minor allele of rs1803274, insecticide exposure increased the risk of PD (Fig. 1b; *P* = 0.008, OR = 2.36, 95% confidence interval: 1.26–4.50) in comparison to the unexposed group with the same genotype. Herbicide exposure led to a strongly elevated PD risk independent of genotype (Fig. 1c).

## Discussion

Studying 24 SNPs within 15 genes involved in pesticide detoxification and transport, we found one SNP (rs1803274 within *BCHE*) that is associated with increased risk for PD in pesticide-exposed Egyptians. *BCHE* codes for the protein butyrylcholinesterase (BChE) that is alternatively designated as pseudocholinesterase or plasma (choline) esterase. The minor allele of rs1803274 defines the K-variant (Kalow variant) of *BCHE*^21^, which has been shown to reduce the activity of functional BChE in serum by 33%^22,23^.

Similar to acetylcholinesterase (AChE), BChE hydrolyses choline esters, e.g. the neurotransmitter acetylcholine (ACh). BChE is 10-fold more common in the body than AChE, yet it does not have unique physiological functions that cannot be compensated by other enzymes. It does, however, play an important role as a bioscavenger protecting against organophosphate and carbamate toxicity^24^. These pesticides prevent degradation of ACh thus causing its accumulation and overstimulation of nerves and muscles with resulting toxic effects^25^. By binding to pesticides, BChE reduces the amount of active substances that can interfere with AChE to induce acute toxicity, or other esterases (e.g. neuropathy target esterase) to induce chronic neurotoxicity^26^.

In our study, insecticides but not herbicides significantly increased PD risk in carriers of the K-variant of *BCHE*. Insecticides used in Egypt are mainly organophosphates (e.g. chlorpyriphos) and carbamates (e.g. carbofuran) that are insufficiently “bioscavenged” by the K-variant of *BCHE* presumably explaining the observed increased risk for PD. In contrast, herbicides are mainly pyrimidines (e.g. bispyribac) and organochlorines (e.g. acetochlor) that do not interact with BChE.

One previous study reported an increased number of individuals with homozygosity for K-variant *BCHE* among PD patients compared to age-matched controls (*P* = 0.051)^27^. This finding, however, has not been confirmed so far^28^. In our sample, K-variant *BCHE* was also not associated with an increased risk for PD by itself, but it possibly facilitated pesticide-induced development of PD owing to the reduced activity and thus less effective bioscavenging property of the K-variant *BCHE*.

Another SNP within *BCHE*, i.e. rs1126680, decreased PD risk in both, pesticide-exposed or unexposed individuals. This is not surprising since rs1126680 does not affect activity and function of BChE even in organophosphate pesticide exposed individuals^29^. Our findings on BChE, however, are not contradictory. In fact they highlight the different roles and functions of BChE under various conditions. On the one hand, BChE acts as a bioscavenger under pesticide-exposed conditions, backing AChE and protecting the brain against toxic effects^26^. However, it has recently been discovered that BChE has its own physiological role affecting brain homeostasis^30^. More important, recent studies proved that BChE might play certain roles in neurodegenerative diseases pathology^31^. However, the functional effects of *BCHE* rs1126680 in this context are unknown so far, but should be elucidated in future investigations.

Since we did not actively match the PD and control groups for age, we assessed the influence of the factors pesticide exposure, coffee consumption, age, and age × pesticide interaction by logistic regression analysis. This approach confirmed pesticide exposure to increase the risk for PD (Table 2), which is consistent with previous observations in different populations^2,5^. Our estimate for pesticide exposure (OR = 7.09, 95% confidence interval: 1.12–44.01) is at the upper end of the range reported in prior studies (OR range: 1.5–7.0)^6^ for pesticide exposure as risk factor of PD but the large confidence interval suggests a high degree of uncertainty concerning the exact value. Studying the efficacy of protective measures in participants working with pesticides in agriculture, we found that wearing gloves during work and washing hands after work reduced the risk for PD (Table 3). This is in line with a previous study showing that glove use and hygiene habits are able to reduce the risk of PD associated with certain pesticides^32^. Additionally, there is convincing evidence that the hands are the most contaminated anatomical region among people working with pesticides^33^. Also, it was shown that different pesticides are rapidly absorbed by the skin^34^ emphasizing that glove use can protect from direct pesticide exposure and thus the risk to develop PD.

Furthermore, we found the well-established protective effect of coffee against PD in the present study as well. In contrast to previous reports, however, we did not find a protective effect of tobacco smoking. This might be due to a possible pesticide contamination of tobacco products in Egypt^26^.

We also identified a higher rate of illiteracy among PD patients as compared to controls. This is consistent with a previous door-to-door study in an Egyptian governorate that revealed a crude prevalence rate of PD of 1,103/100,000 among illiterates, as opposed to 557/100,000 in the general population^18^. Such correlation has not been found in other studies that were mainly conducted in highly industrialized nations. Given that illiteracy was collinear to pesticide exposure in our study, a high degree of illiteracy in pesticide-exposed peasants and less strict adherence to safety measures in this poorly educated group might partially explain the increased risk of illiteracy in Egyptian PD patients. Furthermore, the previously described increased risk for PD in people drinking well water was collinear with pesticide exposure. Therefore, illiteracy and well water drinking are most likely indicators for pesticide exposure in our sample.

The present study confirms pesticide exposure as a risk factor and coffee consumption as a protective factor for PD in an Egyptian population. rs1126680 in *BCHE* decreased the risk for PD regardless of pesticide exposure, and rs1803274 in *BCHE* (K-variant) increased the risk for PD in individuals exposed to pesticides, particularly to insecticides, such as organophosphates and carbamates. This finding provides a basis to identify persons at risk for individualized preventive measures.

## Methods

### Ethics approval

The present study was approved by the ethics committee of Mansoura University, Egypt and the Technical University of Munich, Germany and conducted in accordance with the Declaration of Helsinki and all relevant guidelines and regulations. All study subjects provided written informed consent.

### Study population

PD patients and controls without neurodegenerative disease were enrolled between January 2013 and December 2015 from the collaborating Neurology Departments of the Universities Mansoura, Ain Shams, Assiut, Sohag, Tanta and Zagazig. Participants underwent a standardized clinical assessment by consultant neurologists specialized in movement disorders. Patients with PD were diagnosed using the UK Brain Bank Criteria^35^. Patients with atypical, secondary or familial forms of Parkinsonism or other neurodegenerative diseases were excluded. The modified Hoehn & Yahr stage was ascertained in the on-medication state. Controls without neurodegenerative diseases, as ascertained by history and neurological examination, were recruited from attendants of the collaborating hospitals (healthy visitors or patients without neurodegenerative diseases).

### Questionnaire data collection

Data about environmental factors assumed to modify the risk of PD was collected by trained study assistants in structured interviews using a standardized questionnaire. The questionnaire included the following sections: General information (sex, age, date of birth, ethnicity), disease history (year of diagnosis, disease duration, medication, family history), residence history (duration of rural or urban living), education (literacy, years of education), occupation history (occupation learnt, working history), nutrition habits (coffee, black tea), smoking habits (years and quantity of smoking, cigarette or shisha use), pesticides used at home or at work (duration, frequency, type of pesticides (insecticides, fungicides, herbicides), and pesticide handling (safety precautions, hygienic measures). Some items of the questionnaire were adapted from the risk factor questionnaires of the National Institute of Neurological Disorders and Stroke (NINDS, www.commondataelements.ninds.nih.gov/pd.aspx#tab=Data-Standards). Other factors were added to the questionnaire because of their prior epidemiological association with PD^2,5^. Participants were considered as pesticide-exposed if pesticides were ever used at home or at work or if they resided in a rural area for more than 50% of their lifetime.

### Sample preparation and genotyping

Blood-cell-derived genomic DNA (80–100 ng/µL) was genotyped with the EP1 platform on 96.96 Dynamic Array and read by Fluidigm EP1 Genetic Analysis Scanner (Fluidigm Corporation, San Francisco, CA). Twenty-four Candidate SNPs were chosen in genes related to pesticide detoxification [*CYP1B1* (rs1056836)^36^, *CYP2B6* (rs3745274)^37^, *CYP2C9* (rs1799853, rs1057910)^32^, *CYP2C18* (rs2296680)^38^, *CYP2E1* (rs2070676)^39^, *PON1* (rs662, rs854560, rs854572)^40^, *GSTO1* (rs11191972, rs4925)^41^, *GSTO2* (rs2297235, rs156697)^41^, *NAT1* (rs5030839, rs4987076)^42,43^, *NAT2* (rs15561)^43^, *NQO1* (rs1800566)^9^, *COMT* (rs4680)^36^, *BCHE* (rs1803274, rs1799807, rs1126680)^24,29,44^, *PLA2G6* (CM1211192)^45^] and pesticide transport [*SLC6A3* (rs27072, rs2550956)^14,46^]. Assays include tagged, allele-specific PCR forward-primers and a common reverse primer. Genotypes were determined by using allele specific fluorescent probes (FAM and HEX), which were detected by the EP1 scanner. Data was analyzed by the Fluidigm SNP Genotyping Analysis Software to obtain genotype calls. Automatic calls that did not appear clear were either amended manually or uncalled. The overall call confidence was ≥98.5%.

### Quality control of genetic data

Genetic data quality control was performed using PLINK 1.9 (www.cog-genomics.org/plink1.9/). First all samples were excluded in which >10% of genotypes were not reliably determined. Subsequently all markers that could not be genotyped in >10% of samples, markers with a minor allele frequency <1% and markers with a Hardy-Weinberg *P*-value < 0.0001 were removed. Quality control of genetic data led to a reduction in sample size from 861 in the analysis of demographic and environmental data to 766 samples included in the analysis of genetic data.

### Statistical analysis

Statistical analyses were performed with the statistics software R version 3.3.3 (www.r-project.org). Numbers of study participants in Table 1 were obtained by tabulation. Numbers of study participants positive/negative for more than one variable as found in the results section were calculated by cross-tabulation. Most data were analyzed using logistic regression analyses with models specified in the results section. Logistic regression was performed in base R using the “glm” command, the family “binomial”, and the link function “logit”. Interaction of variables was analyzed by introducing interaction terms into the logistic regression analysis for the parameters indicated in the results section. *P*-values for interactions are if not stated otherwise the *P*-values of the interaction term. To estimate the *P*-value and odds-ratios for pesticide exposure in the rs1803274 wildtype (wt/wt) and variant carriers (wt/var and var/var) we stratified the sample according to these genotypes and performed a logistic regression for the independent variable pesticide exposure. For demographic and environmental variables, a *P*-value of <0.05 was considered significant. For genetic variables, a *P*-value of <0.01 was regarded as significant. A formal correction for multiple testing was not performed because all analyzed variants were candidate variants already described in the context of pesticide exposure by previous studies.

## Electronic supplementary material

Supplementary Information

## Data Availability

The data that support the findings of this study are available from the corresponding author.

## References

[1] Disease GBD, Injury I, Prevalence C (2016). Global, regional, and national incidence, prevalence, and years lived with disability for 310 diseases and injuries, 1990-2015: a systematic analysis for the Global Burden of Disease Study 2015. Lancet.

[2] Ascherio A, Schwarzschild MA (2016). The epidemiology of Parkinson’s disease: risk factors and prevention. Lancet neurology.

[3] Langston JW, Irwin I, Langston EB, Forno LS (1984). 1-Methyl-4-phenylpyridinium ion (MPP+): identification of a metabolite of MPTP, a toxin selective to the substantia nigra. Neuroscience letters.

[4] Khan SU, Lee KS (1976). Determination of cyperquat (1-methyl-4-phenylpyridinium chloride) residues in soil by gas-liquid chromatography. Journal of agricultural and food chemistry.

[5] Bellou V, Belbasis L, Tzoulaki I, Evangelou E, Ioannidis JP (2016). Environmental risk factors and Parkinson’s disease: An umbrella review of meta-analyses. Parkinsonism & related disorders.

[6] Ahmed H, Abushouk AI, Gabr M, Negida A, Abdel-Daim MM (2017). Parkinson’s disease and pesticides: A meta-analysis of disease connection and genetic alterations. Biomed Pharmacother.

[7] Fitzmaurice AG, Rhodes SL, Cockburn M, Ritz B, Bronstein JM (2014). Aldehyde dehydrogenase variation enhances effect of pesticides associated with Parkinson disease. Neurology.

[8] Elbaz A (2004). CYP2D6 polymorphism, pesticide exposure, and Parkinson’s disease. Annals of neurology.

[9] Fong CS (2007). Pesticide exposure on southwestern Taiwanese with MnSOD and NQO1 polymorphisms is associated with increased risk of Parkinson’s disease. Clinica chimica acta; international journal of clinical chemistry.

[10] Paul KC (2016). Organophosphate Pesticide Exposures, Nitric Oxide Synthase Gene Variants, and Gene-Pesticide Interactions in a Case-Control Study of Parkinson’s Disease, California (USA). Environmental health perspectives.

[11] Kiyohara C (2013). MDR1 C3435T polymorphism and interaction with environmental factors in risk of Parkinson’s disease: a case-control study in Japan. Drug Metab Pharmacokinet.

[12] Goldman SM (2012). Genetic modification of the association of paraquat and Parkinson’s disease. Movement disorders: official journal of the Movement Disorder Society.

[13] Manthripragada AD, Costello S, Cockburn MG, Bronstein JM, Ritz B (2010). Paraoxonase 1, agricultural organophosphate exposure, and Parkinson disease. Epidemiology.

[14] Ritz BR (2009). Dopamine transporter genetic variants and pesticides in Parkinson’s disease. Environ Health Perspect.

[15] Biernacka JM (2016). Genome-wide gene-environment interaction analysis of pesticide exposure and risk of Parkinson’s disease. Parkinsonism Relat Disord.

[16] Mansour SA (2004). Pesticide exposure–Egyptian scene. Toxicology.

[17] Rohlman DS (2014). Characterizing exposures and neurobehavioral performance in Egyptian adolescent pesticide applicators. Metab Brain Dis.

[18] Khedr EM (2012). Epidemiological study and clinical profile of Parkinson’s disease in the Assiut Governorate, Egypt: a community-based study. Neuroepidemiology.

[19] Khedr EM (2015). Prevalence of Parkinsonism and Parkinson’s disease in Qena governorate/Egypt: a cross-sectional community-based survey. Neurol Res.

[20] Benamer HT, de Silva R, Siddiqui KA, Grosset DG (2008). Parkinson’s disease in Arabs: a systematic review. Movement disorders: official journal of the Movement Disorder Society.

[21] La DBN, Bartels CF, Nogueira CP, Arpagaus M, Lockridge O (1991). Proposed nomenclature for human butyrylcholinesterase genetic variants identified by DNA sequencing. Cellular and molecular neurobiology.

[22] Rubinstein HM, Dietz AA, Lubrano T (1978). E1k, another quantitative variant at cholinesterase locus 1. Journal of medical genetics.

[23] Maetzler W (2009). No differences of butyrylcholinesterase protein activity and allele frequency in Lewy body diseases. Neurobiology of disease.

[24] Lockridge O, Norgren RB, Johnson RC, Blake TA (2016). Naturally Occurring Genetic Variants of Human Acetylcholinesterase and Butyrylcholinesterase and Their Potential Impact on the Risk of Toxicity from Cholinesterase Inhibitors. Chem Res Toxicol.

[25] Sanchez-Santed F, Colomina MT, Herrero Hernandez E (2016). Organophosphate pesticide exposure and neurodegeneration. Cortex.

[26] Read DJ, Langford L, Barbour HR, Forshaw PJ, Glynn P (2007). Phospholipase B activity and organophosphorus compound toxicity in cultured neural cells. Toxicology and applied pharmacology.

[27] Singleton AB, Gibson AM, Edwardson JA, McKeith IG, Morris CM (1998). Butyrylcholinesterase K: an association with dementia with Lewy bodies. Lancet.

[28] Chang D (2017). A meta-analysis of genome-wide association studies identifies 17 new Parkinson’s disease risk loci. Nat Genet.

[29] Howard TD (2010). Evaluation of candidate genes for cholinesterase activity in farmworkers exposed to organophosphorus pesticides: association of single nucleotide polymorphisms in BCHE. Environmental health perspectives.

[30] Brimijoin S, Chen VP, Pang YP, Geng L, Gao Y (2016). Physiological roles for butyrylcholinesterase: A BChE-ghrelin axis. Chemico-biological interactions.

[31] DeBay DR (2017). Butyrylcholinesterase-knockout reduces fibrillar beta-amyloid and conserves (18)FDG retention in 5XFAD mouse model of Alzheimer’s disease. Brain research.

[32] Furlong M (2015). Protective glove use and hygiene habits modify the associations of specific pesticides with Parkinson’s disease. Environ Int.

[33] Atabila A (2017). Dermal exposure of applicators to chlorpyrifos on rice farms in Ghana. Chemosphere.

[34] Berankova M, Hojerova J, Melegova L (2017). Exposure of amateur gardeners to pesticides via the non-gloved skin per day. Food Chem Toxicol.

[35] Hughes AJ, Daniel SE, Kilford L, Lees AJ (1992). Accuracy of clinical diagnosis of idiopathic Parkinson’s disease: a clinico-pathological study of 100 cases. Journal of neurology, neurosurgery, and psychiatry.

[36] Ghisari M, Long M, Bonefeld-Jorgensen EC (2013). Genetic polymorphisms in CYP1A1, CYP1B1 and COMT genes in Greenlandic Inuit and Europeans. Int J Circumpolar Health.

[37] Dadson OA (2013). Metabolism of profenofos to 4-bromo-2-chlorophenol, a specific and sensitive exposure biomarker. Toxicology.

[38] Koutros S (2011). Xenobiotic-metabolizing gene variants, pesticide use, and the risk of prostate cancer. Pharmacogenet Genomics.

[39] Shahabi HN (2009). Cytochrome P450 2E1 gene polymorphisms/haplotypes and Parkinson’s disease in a Swedish population. J Neural Transm (Vienna).

[40] Belin AC (2012). Association of a protective paraoxonase 1 (PON1) polymorphism in Parkinson’s disease. Neuroscience letters.

[41] Kiyohara C (2010). GST polymorphisms, interaction with smoking and pesticide use, and risk for Parkinson’s disease in a Japanese population. Parkinsonism & related disorders.

[42] Potts LF (2012). Polymorphic genes of detoxification and mitochondrial enzymes and risk for progressive supranuclear palsy: a case control study. BMC Med Genet.

[43] Zhu Y, States JC, Wang Y, Hein DW (2011). Functional effects of genetic polymorphisms in the N-acetyltransferase 1 coding and 3’ untranslated regions. Birth Defects Res A Clin Mol Teratol.

[44] Kurdyukov I, Rodionov G, Radilov A, Babakov V (2014). Genotyping single-nucleotide polymorphisms of human genes involved in organophosphate detoxification by high-resolution melting. Anal Bioanal Chem.

[45] Gui YX (2013). Four novel rare mutations of PLA2G6 in Chinese population with Parkinson’s disease. Parkinsonism & related disorders.

[46] Xu M (2010). Pharacogenetic effects of dopamine transporter gene polymorphisms on response to chlorpromazine and clozapine and on extrapyramidal syndrome in schizophrenia. Progress in neuro-psychopharmacology & biological psychiatry.

