# Supplementary material for: K-variant BCHE and pesticide exposure: Gene-environment interactions in a case–control study of Parkinson’s disease in Egypt
Source: Sci Rep. 2018 Nov 8;8:16525. doi: 10.1038/s41598-018-35003-4 (PMC6224461; doi:10.1038/s41598-018-35003-4)
Supplement: Supplementary file 1 — Supplementary Information [file 41598_2018_35003_MOESM1_ESM.pdf]

K-variant BCHE and pesticide exposure: Gene-environment interactions in a case-control study of Parkinson's disease in Egypt

Thomas W. Rösler, Mohamed Salama, Ali S. Shalash, Eman M. Khedr, Abdelhalim El-Tantawy, Gharib Fawi, Amal El-Motayam, Ehab El-Seidy, Mohamed El-Sherif, Mohamed El-Gamal, Mohamed Moharram, Mohammad El-Kattan, Muhammad Abdel-Naby, Samia Ashour, Ulrich Müller, Astrid Dempfle, Gregor Kuhlenbäumer & Günter U. Höglinger

Supplementary Table S1. Results for all SNPs in the logistic model.

SNP	pSNP	OR-SNP(95%CI)	pSNP*EX	OR-SNP*EX(95%CI)
rs1056836	0.615	1.119(0.723-1.735)	0.695	0.871(0.436-1.735)
rs1803274	0.187	0.753(0.493-1.146)	0.007	2.568(1.298-5.128)
rs1799807	0.212	0.633(0.303-1.285)	0.737	1.238(0.354-4.326)
rs1126680	0.002	0.379(0.199-0.695)	0.795	0.89(0.369-2.157)
rs27072	0.876	0.966(0.621-1.5)	0.154	0.598(0.294-1.212)
rs2550956	0.129	1.441(0.9-2.315)	0.134	0.563(0.265-1.193)
rs662	0.115	0.72(0.479-1.082)	0.12	1.681(0.874-3.237)
rs854560	0.647	0.91(0.605-1.364)	0.701	0.88(0.457-1.691)
rs854572	0.957	1.012(0.659-1.555)	0.282	0.682(0.337-1.368)
rs4987076	0.542	1.17(0.706-1.938)	0.527	1.312(0.568-3.064)
rs5030839	0.123	0.413(0.123-1.217)	0.73	1.414(0.187-10.272)
rs15561	0.178	1.325(0.881-1.999)	0.369	1.363(0.694-2.687)
rs2296680	0.593	1.138(0.708-1.829)	0.268	1.541(0.72-3.331)
rs1799853	0.672	0.894(0.532-1.497)	0.524	1.291(0.589-2.845)
rs1057910	0.15	0.627(0.327-1.174)	0.405	1.661(0.505-5.605)
rs11191972	0.565	1.127(0.75-1.695)	0.392	0.752(0.391-1.445)
rs4925	0.56	1.128(0.753-1.691)	0.451	0.78(0.408-1.489)
rs2297235	0.688	1.089(0.717-1.655)	0.551	0.816(0.418-1.593)
rs156697	0.194	1.347(0.861-2.119)	0.258	0.658(0.317-1.357)
rs2070676	0.171	0.753(0.501-1.129)	0.632	0.853(0.443-1.636)
rs1800566	0.202	0.766(0.507-1.152)	0.296	0.706(0.366-1.356)
rs3745274	0.103	0.712(0.473-1.069)	0.315	1.395(0.728-2.675)
rs4680	0.603	0.889(0.571-1.384)	0.65	1.18(0.576-2.413)
rs1056836	0.615	1.119(0.723-1.735)	0.695	0.871(0.436-1.735)

Logistic regression analysis of SNP main effect and interaction with pesticides. The interaction *P*-value is derived from the interaction term between SNP and pesticide exposure. The logistic model contained SNP, pesticide exposure, age, coffee consumption and the interaction between pesticides and SNP as well as pesticides and age; affection-status ~ SNP + pesticide-exposure + coffee + age + (SNP * pesticide-exposure) + (age * pesticide exposure). pSNP: p-value for association between SNP and PD, OR-SNP(95%CI): corresponding odds ratio for the association between SNP and PD, pSNP*EX: p-value for the interaction between SNP and exposure, OR-SNP*EXP(95%CI): corresponding odds ratio for the interaction between SNP and exposure. This odds ratio applies only to the interaction but not to the PD risk conferred by the joint action of SNP and exposure. For the latter see Figure 1.
